# Hidden work and blurred boundaries: a qualitative study of how community nurses navigate and adapt injectable medication processes to provide timely end-of-life symptom control

**DOI:** 10.1186/s12904-026-02159-0

**Published:** 2026-05-28

**Authors:** Rosanna Fennessy, P. John Clarkson, Ben Bowers

**Affiliations:** 1 Primary Care Unit, Department of Public Health and Primary Care, University of Cambridge, Cambridge, CB2 0SR UK; 2 Department of Engineering, University of Cambridge, Cambridge, UK

**Keywords:** Community nursing, End of life care, Anticipatory prescribing, General practitioners, Community health nursing, Qualitative methods

## Abstract

**Background:**

With rising numbers of people dying at home, often with complex multimorbidity, community healthcare professionals are increasingly responsible for providing end-of-life care. This care is usually led by community nurses, many of whom do not have specialist palliative care training. A core part of their role is providing end-of-life symptom control using injectable medications prescribed ahead of need and stored in patients’ homes. However, cross-organisational systems around injectable medication processes are complex and time consuming. We aimed to explore community nurses’ perceptions of their changing roles in end-of-life care and how they navigate injectable medication processes to provide timely symptom control.

**Methods:**

A qualitative study using in-depth interviews with community nurses undertaken in two counties in England. Data were analysed using reflexive thematic analysis.

**Results:**

Fifteen community nurses took part. Nurses’ roles in end-of-life care were characterised by increasing scope and complexity, making it challenging to offer the holistic care they wanted to. Using their practical knowledge and wisdom, nurses navigated and sometimes adapted (changed) challenging injectable medication processes to ensure timely symptom control for patients. This involved considerable additional hidden work conducted on behalf of patients, families, nursing colleagues and general practitioners. Some nurses appreciated the autonomy and independence they had in navigating and adapting injectable medication processes. However, others perceived their expanding remit in end-of-life care and the increasingly blurred boundaries between their role and that of general practitioners as sources of anxiety and professional risk.

**Conclusions:**

To ensure community nurses feel confident, empowered and professionally safeguarded to take on this multifaceted and increasingly complex work, further experiential learning with palliative care colleagues is required, in addition to assured support from regulatory organisations. Exploring how other healthcare professionals, including pharmacists and paramedics, alongside families, navigate and adapt injectable medication care can make other hidden work visible and identify priority areas for system-wide improvements.

**Supplementary Information:**

The online version contains supplementary material available at 10.1186/s12904-026-02159-0.

## Background

Globally, the majority of deaths occur outside hospital settings [1]. In the UK, the Covid 19 pandemic prompted an increase in the number of deaths in community settings, and this trend has continued [2–4]. Patients requiring complex end-of-life care are increasingly being managed by community healthcare professionals, including community nurses and general practitioners (GPs) [5, 6]. Different kinds of nurses support families at the end-of-life in the community, most of whom are not specialists in palliative care (Table 1). Nevertheless, they provide core end-of-life care alongside their other clinical activities, including chronic disease care and wound management.

**Table 1:** Community-based clinicians primarily involved in injectable medication care in the UK

Role Definition	
Community nurses	*Registered nurses who provide care for adults in their own homes or residential care homes. This includes nurses with the additional District Nursing Specialist Practitioner Qualification.*
Palliative care nurses	*Registered nurses specialised in supporting patients with complex*,* life-limiting conditions and illnesses.*
Nurse prescribers	*Registered nurses who have completed an independent non-medical prescribing qualification. The types of medications which may be prescribed vary according to the type of prescribing qualification.*
General Practitioners (GPs)	*Family doctors who oversee and provide general medical care*,* usually in the community.*
Paramedics	*Registered healthcare professionals who provide emergency clinical care to acutely unwell or injured patients*,* including in the community.*
Palliative care doctors	*Physicians specialised in supporting patients with complex*,* life-limiting conditions and illnesses.*

Although the demand for community end-of-life care is increasing, globally there are challenges with nurse retention and recruitment as a result of increased workload and job strain [7–9]. For example, between 2009 and 2024, the number of district nurses in England declined by 43% [10, 11]. Community end-of-life care has also been impacted by changes in GP working practices, including more remote working such as phone or video calls replacing in-person home visits. This means GPs are increasingly looking to community nursing teams and specialist palliative care nurses to lead and provide complex assessments and symptom management interventions [12–15].

A key community end-of-life care intervention is the prescription of injectable symptom control medications, either ahead of need (anticipatory prescribing) or in response to existing symptoms [13, 16, 17]. This is considered best practice in many countries to alleviate distressing end-of-life symptoms such as pain, breathlessness, agitation and respiratory secretions in a timely manner, when oral routes are no longer viable [18, 19]. Community nurses typically inform GPs when injectable medication prescriptions are required [13], and once prescribed, they are usually collected by family members and kept in the patient’s home along with preapproved ‘permission to administer charts’ (standing orders). These charts allow visiting community nurses and paramedics to administer injectable medications over a range of specified dosages [17]. However, systems for prescribing and using injectable medications can be complex and involve multiple actors and care providers working together, often in stressful and changing circumstances [20]. There can be recurring challenges such as sourcing timely clinical assessments, prescriptions and valid permission to administer charts, obtaining medication supplies and ensuring timely administration [16, 21, 22].

Amid rising caseloads and increasing patient complexity, there remains limited understanding of how community nurses manage and navigate suboptimal injectable medication systems to ensure timely end-of-life care for patients, and how these experiences interact with their perceptions of their role and professional identity.

## Methods

### Study aim

We aimed to explore community nurses’ perceptions of their changing roles in end-of-life care and how they navigate and manage injectable medication processes to provide timely end-of-life symptom control.

### Study design

This paper reports findings from a qualitative analysis of interviews with community nurses as part of a larger longitudinal, multi-perspective study which also focuses on patient and carer experiences of the prescribing and use of anticipatory medications. The study is based around sixteen patient ‘cases’. A patient case comprised adult patients living at home who had been prescribed injectable end-of-life symptom control medications. Interviews were undertaken with the patients and/or their family carers and healthcare professionals involved in their care.

The London Camberwell St Giles Research Ethics Committee approved the study [Reference number: 23/LO/0984].

We used the Standards for Reporting Qualitative Research (SRQR) guidelines when drafting and editing this manuscript [23].

### Patient and public involvement

We had regular in-person and online meetings throughout the project with our Public and Clinician Advisory Group, comprising four family carers and two practicing community nurses with lived experience of injectable medication care. They were involved with study design and refining the topic guides. They also provided invaluable analytical insights into our findings and implications for practice. Patient and Public Involvement (PPI) is reported using the Guidance for Reporting Involvement of Patients and the Public (GRIPP2) checklist [24].

### Setting and recruitment

The study took place in two counties in England covering urban, rural and coastal populations with varying levels of economic deprivation. Patients or their family carers who were taking part in the study, were asked to nominate up to three community-based healthcare professionals who had been involved in their injectable medication care. These professionals could have worked for any of the fourteen NHS healthcare organisations taking part in the research. Nominated individuals were sent a research invitation letter by the study’s lead in their organisation. Details of interested healthcare professionals were passed to the research team who contacted them with further information. Only nurses expressed an interest in taking part. Participants provided written informed consent before their one-off interviews. All were offered a £40 gift voucher for their time.

### Data collection

In-depth semi-structured interviews took place between May and December 2024, with purposive sampling informed by the principles of information power [25]. A range of community nurses and palliative care nurses across regions, levels of experience and seniority were purposively recruited. Interviews were conducted by phone or Teams video call by an experienced clinical academic community nurse (BB) or an experienced non-clinical research psychologist (RF). The topic guide explored nurses’ perceptions of their role in end-of-life care and their experiences related to navigating cross-organisational systems for prescribing and using injectable medications (Supplementary material file 1). The interviews also included questions related to specific patient cases, but these data were not analysed in the present study.

Interviews lasted between 35 and 72 min. These were audio recorded, professionally transcribed verbatim, and anonymised prior to analysis. All names used are pseudonyms.

### Data analysis

#### Analysis approach and reflexivity

Our study adopted a constructivist approach which assumes that individuals’ unique perspectives of injectable medications and end-of-life care are shaped by their social interactions with others and experiences over time [26]. We acknowledged the influence of our own experiences and subjectivity during data collection and analysis, engaging in reflexive practices such as extended written reflections and collaborative sense-making throughout the study. Semantic coding and analysis of data followed a thematic and constant comparison approach, looking for patterns and divergences amongst participants’ experiences [27]. For example, as well as noting similarities within shared narratives, we also looked for differences in perspectives, including those potentially influenced by nurses’ level of experience, local ways of working, palliative care expertise and non-specialist / specialist roles.

#### Analysis steps

- All transcripts were read through by RF and BB for data familiarisation.
- Three were selected at random and coded inductively by RF noting points of interest, similarity and difference. Discussion and reflection with BB resulted in an agreed analytical focus on the different aspects of community nurses’ roles and how nurses managed the work of timely injectable medication care.
- RF inductively coded all transcripts in NVivo 14, using constant comparison techniques to compare experiences by nursing roles and perceived expertise; themes were constructed around how nurses perceived the extent of their roles and how they navigated and adapted injectable medication care to support patients and others.
- Initial analytical themes were shared with our Public and Clinician Advisory Group during an in-person meeting. Group discussions helped inform the iterative analysis, ensuring it was both relevant and useful in terms of lived experiences and implications for practice.

## Results

Fifteen nurses took part. Participants included community nurses (who were not specialists in palliative care) and specialist palliative care nurses with a range of professional experience, years of service and pay bands (Table 2). The eleven community nurses working in district nursing teams reported having considerable in-person patient contact. The four palliative care nurses provided both in-person and telephone-based care. Participants worked across multiple teams within five different organisations, with representation in both English regions.

**Table 2:** Participant characteristics

Role	Gender	Ethnicity	Years qualified	Pay Band ^a^	Non-medical Prescriber
Community nurses ^b^ (*n* = 11)	*10 Female* *1 Male*	*9 White British* *1 White European* *1 British Asian*	*2 (> 1 year)* *1 (2-5yrs)* *4 (6-10yrs)* *4 (10 + yrs)*	*Six Band 5* *Four Band 6* *One Band 7*	*2* ^*c*^
Palliative care nurses (*n* = 4)	*4 Female*	*4 White British*	*0 (> 1 year)* *0 (2-5yrs)* *2 (6-10yrs)* *2 (10 + yrs)*	*One Band 5* *Three Band 7*	*2*

^a^ In the UK, pay band 5 nurses are staff nurses and bands 6 and 7 are more senior nurses with both patient caseload and team management responsibilities

^b^ Community nurse roles included community staff nurse (n=6), community sister (n=4), community nurse clinical lead (n=1)

^c^ One non-medical prescriber participant was unable to prescribe end-of-life injectable medications due to local policy restrictions

Nurses’ perceptions of their role in end-of-life care and how they navigated systems to provide timely symptom control were constructed into three interconnected themes:

- Core and expanding roles in end-of-life care.
- Hidden work and role ambiguity.
- Varying comfort with expanding roles and adapting systems.

### Core and expanding roles in end-of-life care

Participants saw their core role in end-of-life care in the community as providing timely symptom management and support for patients. Ensuring that injectable medications were prescribed, dispensed and available to use when necessary was seen as fundamental to their role in ensuring patients had a comfortable, dignified death:

*The best way that somebody can potentially die*,* that’s what matters the most to me*,* that they’re not in pain*,* they’re not anxious*,* they’re not full of audible secretions… so it’s being there for the patient and their loved ones in a consistent manner which obviously means medications and making sure that they’re fully supported. [Rachel*,* community staff nurse]*

In addition to providing symptom management input, nurses also discussed other aspects of their roles in delivering personalised end-of-life care. These included building relationships with patients and families, helping them manage changing situations and needs, organising equipment or carers, and offering psychological support. However increasing caseloads and patient complexity often made it difficult to offer holistic care:

*Patients have become more complex*,* so visits that used to last 10–15 min*,* we’re now going in and there seems to be a lot more wrong with people*,* so visits are lasting longer*,* but you don’t have any more hours in your day to do them. Compared to 20 years ago we’d see 7 or 8 patients in a day*,* we’re now seeing 22*,* 23. [Mandy*,* community staff nurse]*

Whilst all nurses valued their core role in offering end-of-life symptom management support, some community nurses without specialist palliative care training reported that additional roles that were increasingly part of their clinical practice presented challenges. In particular, taking the lead in explaining injectable medications, co-ordinating highly complex care and spending extended periods of time supporting patients were perceived as demanding. Some community nurses rationalised that they were not experts in end-of-life care and expanding the boundaries of their palliative care input caused additional pressures for them:

*I think also*,* because as district nurses we kind of have lots of fingers in lots of pies*,* we don’t just do palliative care*,* we do catheter care*,* diabetic care*,* wound care*,* we’re not really experts in palliative care and if it’s something out of the norm*,* it can be a lot of pressure. [Ava*,* community sister]*

### Hidden work and role ambiguity

To ensure timely symptom control for patients and manage challenges with injectable medication prescribing and administration processes, nurses used their practical knowledge and experience to both work within (navigate) and sometimes change (adapt) existing systems. These activities were individually instigated by nurses and demonstrated considerable hidden work in end-of-life care. Nurses adapted to situations and completed tasks typically undertaken by GPs or patients’ families, highlighting they were working with increased role ambiguity, including where the professional boundary of a nurse ends and a GP begins.

Nurses shared how they planned ahead on behalf of patients and colleagues. They had learned through experience of the detrimental impact of limited staffing and stretched community healthcare systems on providing timely injectable medication care. Several participants described using their clinical judgement to predict a patient’s likely deterioration ahead of a weekend and they asked GPs to prescribe syringe drivers in advance, to prevent delays in sourcing medical input and prescriptions during out-of-hours periods:

*…and being a Friday*,* I think I spoke with the GP to kind of consider prescribing or writing him a MAR [permission to administer] chart for the syringe driver as well*,*…. I thought maybe think ahead*,* should Alan need a lot of stat doses*,* he will benefit*,* … because*,* you know*,* despite all our best efforts to come in as soon as we can*,* you know*,* we’re not going to be able to be there*,* instantly*,* because that’s just limitations*,* and we have to be mindful of that as well. [Justin*,* community staff nurse]*

Similarly, where nurses considered that colleagues coming onto the next shift might be short of supplies, they instigated replenishments including prescriptions, permission to administer charts, medications, syringes, water for injection, and even batteries for syringe pumps. To secure medication supplies, nurses described giving GPs a ‘nudge’ to renew prescriptions and accompanying charts with suitable titrated dose ranges before they were needed, to prevent delays in timely symptom control:

*Because I work shifts*,* I do 12 h days*,* if I’m not on the next day I don’t want to leave someone else to have to chase where the meds are*.* If I know that the prescription’s been done and sent electronically to the pharmacy then I’m happy*,* and I know they’ve got stock*,* and in my mind that’s kind of hopefully fail-safe. [Rachel*,* community staff nurse]*

Families are typically expected to collect medications and permission to administer charts once prescribed. However, it is not uncommon for medications to be unavailable or subject to delays when ordered via a patient’s nominated pharmacy. In these cases, several nurses reported intervening on behalf of families; they undertook considerable hidden administrative work by contacting various pharmacies to see who held stock. They would then ask GPs to send new prescriptions to that pharmacy.

*I don’t know that everyone does this*,* but what I do is ring round the chemists*,* find out who’s got them in stock*,* I know what I want and what I need*,*. and I will ask the GP to send it to that nominated pharmacist and I’ll say on my request that I’ve checked*,* they’ve got them in stock*,* please send the prescription there. [Amy*,* community staff nurse]*

While nurses’ efforts to chase medications around different pharmacies might be successful during the day, the poor availability of medications during out-of-hours periods could sometimes call for what nurses described as *‘thinking outside the box’*. This included a specialist palliative care nurse contacting paramedics via emergency services as they knew ambulances carried injectable morphine. However, this unofficial adaptation was acknowledged as potentially having a detrimental effect on other patients and wider service provision:

*It’s an emergency service that we’re using for a non-emergency purpose*. [*Ashley, palliative care nurse*]

To ensure prescriptions and accompanying permission to administer charts for patients were arranged in a timely manner, most nurses made a particular point of fostering good working relationships with GP surgeries, pharmacies and other nursing services. This hidden relationship-building work helped them to get rapid advice, support and medication supplies when needed. These relationships often took time to build but were seen as highly beneficial in enabling nurses to carry out their core roles and make in-the-moment adaptations:

*If we’ve built up relationships with the GPs*,* I mean the secretaries that I send an email to request these medicines*,* they know who I am*,* they get it through very quickly*,* same with the district nurses. The GPs again know who I am*,* there’s that relationship*,* they’ll know if I’m asking for medicines I’ll need them and they’ll get it processed quicker. [Amy*,* community staff nurse]*

A recurring problem experienced by all participants were errors or omissions in prescriptions or written directions on permission to administer charts. Nurses attributed these to the increasing caseloads of GPs which meant they had less contact with patients at their end-of-life than in the past, or some GPs unfamiliarity with the drugs and dose ranges required. These errors or omissions led to nurses having to explain to distressed patients and families that they could not administer injectable medications when needed in a crisis:

*Sometimes the GP will incorrectly populate the prescription chart*,* therefore the nurses cannot administer off the chart*,* and then we are standing in a patient’s house*,* who’s in agony*,* and agitated*,* so that can be quite tricky*,* and frustrating for the family members*,* saying*,* “Can’t you just give it*,* because it’s prescribed*,*” but*,* you know*,* just because there’s a technical error*,* and then you’ve got to say*,* “Clinically I cannot do it*,* it’s against the rules*,* because this MAR [permission to administer] chart is actually null and void.” So it’s quite tricky when that happens. [Kiara*,* community staff nurse]*

To try to avoid problems with incorrect prescriptions or charts which impacted nurses’ ability to administer medication, they frequently stepped in to direct GPs, regarding which specific medications and doses to prescribe. Although nurses were aware they were effectively taking on the GP role they were conscious of not offending medical colleagues or usurping colleagues’ authority by being polite and framing their requests as suggestions:

*I mean if I go into a GP and ask them to write it*,* I stand and watch them write it and be like*,* “Oh no can you fill that bit in? You haven’t put that dose right”*,* and they do it there and then with us. [Mandy*,* community staff nurse]*

Some GPs were not keen to take nurses’ advice regarding prescriptions. When dealing with these colleagues, nurses used strategies to add additional authority to their request such as involving specialist palliative care in decision-making:

*I mean the one doctor that I’m thinking about in particular*,* he won’t take advice…I know when I speak to him I put everything on SystmOne [electronic health records] and I speak to palliative care*,* so I know exactly what the person needs and it’s all on SystmOne [Jess*,* community staff nurse]*.

All participants stated that local policies meant they were not able to take medications to patients’ homes or to dispose of them after a patient’s death. While some nurses adhered strictly to this rules-based practice, some experienced nurses commented that where patients did not have a family member, carer or friend who could undertake this task, they would do this despite it being against ‘policy’. Nevertheless, they remained concerned about the potential risk to their professional registration in doing so and looked to mitigate this:

*I’m not supposed to. It’s against policy. That’s the correct answer but I think it depends on the circumstances*,* it depends on the situation*,* you know. If you had a patient whose relative couldn’t get out*,* you know*,* I think if I was ever put in a situation like that I would ring the team and ask and make them aware*,* you know “I’ve been asked to do this*,* what do you want me to do?”. [Grace*,* palliative care nurse]*

### Varying comfort with expanding roles and adapting systems

Participants varied widely in their reported comfort with undertaking hidden work and adaptations to support patients with timely symptom control. For some community nurses, the ambiguity created by the expansion of their role and its blurred boundaries with the work of medical colleagues created worries about overstepping their jurisdiction, and the potential risks for their professional registration.

Adaptations where nurses stepped out of their typical roles were often viewed differently depending on nurses’ level of experience and perceived autonomy. More experienced nurses were comfortable with advising GPs about how to complete prescriptions and charts. This was particularly evident when approaching GPs about disagreements on what drugs and dose ranges should be prescribed, described by one participant as requiring professional ‘*guts*’. For less experienced nurses these encounters could be challenging:

*So some of our nurses are more experienced than others*,* as in more areas*,* some know exactly what needs to go in that driver*,* others not so much and it can make you feel quite uncomfortable when you’ve got a GP wanting you to tell them what they’ve got to write on the chart*,* and where to put it*,* that can be hard going*,* sometimes. [Siobahn*, *community nurse clinical lead]*

The blurring of boundaries of professional roles between nurses and GPs made some participants uncomfortable. They noted that they were not qualified to prescribe, and taking on this additional work was beyond their professional jurisdiction:

…*it took our nurse having to say*,* "no*,* you’ve written that wrong*,* you’ve written that wrong"*,* multiple times to get it right. And like I said*,* we’re not prescribers*,* we shouldn’t be leading that*,* all we should technically have to do is implement the care*,* which is sad*,* really. So*,* yeah*,* we do a lot of GP hand holding. [Ava, community sister]*

Once medications were prescribed, provided there was an accurate permission to administer chart in place, nurses had the professional authority to act in real time without needing to refer to others for permission to start medications and titrate doses. For some community nurses, having this autonomous role was seen as empowering:

*I think that the best part of injectables is the point where the nurses are the primary clinician involved*,* where they are setting how much medication this particular patient needs over a 24-hour period*,* and then they can base their decision to start a continuous syringe driver. [Kiara, community staff nurse]*

However, others were less comfortable with this responsibility, commenting that deciding when to use injectable medications in the community presented significant professional risk. Nurses who were not specialists in palliative care discussed concerns about their Professional Identification Number (PIN) if their authority or decisions were challenged by patients and carers, including in cases where they assessed that patients did not need medications despite families requesting their use.

*There are times when I have felt really pressured into giving medication. You have to sit and think*,* because everything has to be evidence-based and you’ve got to justify your PIN [Professional Identification Number] to that injection*,* and if you give that injection and that patient does pass away soon after*,* it could be coincidence*,* but the family might not see it like that. [Rachel, community staff nurse]*

Community nurses also expressed that dealing with controlled drugs or placing syringe drivers in their lone-working roles meant they faced greater professional risk compared to nurses working in institutional settings where this work is undertaken in pairs. One participant noted that some community nurses in their area, even those with significant experience, were not prepared to renew syringe drivers alone due to anxieties about potential mistakes. This left other nurses feeling pressured to take on this work for their colleagues:

*And you’re on your own generally as well*,* so often you think*,* oh dear you know have I made the right decision? It’s your word against theirs*,* isn’t it*,* really. Because you don’t go out in twos*,* you don’t check anything*,* we’re always out*,* well generally out on our own. [Jess*,* community staff nurse]*

To share and mitigate perceived risks, nurses frequently consulted with, or deferred to others, usually experienced specialist palliative care colleagues. This was also a way to validate their own clinical decisions before using medications:

*So personally*,* I would get advice from a palliative nurse. Because it is a scary thing because you’re on your own*,* you’re on your own making decisions and*,* you don’t want to make the wrong one. But usually*,* you know*,* it’s just having that person going*,* no*,* you’re right*,* they do need pain relief. [Ava*,* community sister]*

A notable example of this deferring or sharing of responsibility was nurse prescribing. Four participants were registered nurse prescribers, but three commented that they did not actively prescribe injectable end-of-life medication unless there was an immediate need. Instead, they preferred the responsibility for writing these prescriptions to remain with the GP, although they were comfortable with advising the GP regarding what medications and doses to prescribe. The participants reasoned it kept the GP involved in these decisions and the paper prescriptions which are used by nurses fail to create a clear audit trail in electronic records:

*Because we don’t have electronic prescribing*,* so if we prescribe anything it’s always done by the FP10 paper copy so it’s not on the electronic system… In a way it’s better if we liaise with the GP*,* ask them to actually physically issue them*,* the drugs*,* which means them prescribing them in effect*,* but we then say*,* "we’ve done the MAR [permission to administer] charts*,* which says this*,* this and this*,* please could you issue the medication". [Casey*,* palliative care nurse]*

However, for some community nurses the idea of being responsible for prescribing was undesirable; particularly as they felt they did not get paid enough to take on the professional risk involved:

*I personally wouldn’t want to become a nurse prescriber because*,* actually*,* I think it’s a lot of ownership on you as a nurse… I also feel*,* if you’re a nurse prescriber you should be paid more …and that’s not going to happen*,* so why do it if you’re not going to get paid more? [Ava*,* community sister]*

## Discussion

Community nurses use their practical knowledge and a range of tactics to navigate and adapt systems for prescribing and using injectable medications to ensure timely symptom control for patients. In an increasingly time-pressured and resource-constrained context of community care, this constitutes significant and largely hidden work for nurses working in England. These hidden forms of work and adaptations also highlight the expanding scope of non-specialist community nurses’ roles, including a blurring of professional boundaries between nurses and GPs. While some of our community nurse participants welcomed the independence and autonomy afforded by taking on this work, many expressed concerns about their levels of palliative care expertise, and potential risks to their professional registration.

The nurses in our study preferred to adopt a holistic approach to end-of-life care; however, they often found it difficult to provide support beyond physical nursing tasks. The inability to provide additional aspects of community nursing such as developing therapeutic relationships with patients and families was attributed to workload pressures and increasingly complex caseloads, mirroring other recent study findings both in England and internationally [8, 10, 28, 29]. Conversely, offering end-of-life care support beyond core physical nursing tasks was a source of anxiety for some nurses when they felt they lacked sufficient expertise. Providing end-of-life care in the community involves working independently and managing uncertainty and potentially difficult conversations [29–31]. The need for increased training and time working with experienced others (experiential learning) to develop confidence and skills has been widely discussed but remains poorly enacted [28, 29, 32]. Our findings also highlight that community nurses require access to support and advice to feel comfortable with their expanding professional jurisdiction in leading and providing complex symptom control and wider end-of-life care.

Challenges with current systems for prescribing and using injectable medications have been widely documented, including avoiding errors in prescriptions and permission to administer charts, sourcing supplies from pharmacies and ensuring timely administration [16, 28]. Our study adds to this literature by illustrating how nurses overcome these challenges by using their practical knowledge and experience of working both within, and adapting, injectable medication systems. However, this requires considerable hidden work to ensure timely symptom support for patients. Community nurses’ hidden work remains under researched since the concept has primarily been applied to nurses in hospitals where their hidden ‘articulation’ work is described as “ensuring actions, people and materials required to support patient care are lined up in the right place at the right time” (Allen, 2015) [33–35]. Our study demonstrates the considerable hidden articulation work also undertaken by community nurses, including anticipating the needs of patients and colleagues in terms of prescriptions, medications and accompanying equipment, advocating for patients, along with time and energy spent developing relationships with GP surgeries and community pharmacies to facilitate timely end-of-life care. Improvements with access to community pharmacies and end-of-life medication, particularly during out of hours periods, would reduce some of this hidden work. Further research investigating the hidden articulation work undertaken by other healthcare professionals such as paramedics and pharmacists, along with families, who are also involved in injectable medication care processes, will provide a useful lens to make visible the range of hidden tasks that enable complex systems to function. This will help in identifying key areas for system-wide improvements to facilitate person-centred care and timely symptom management.

Interactions where nurses directed GPs with what to prescribe to avoid potential errors and ensure appropriate and timely care highlighted the increasingly blurred boundaries between the roles of nurses and GPs in end-of-life care. This practice has also been noted in other studies [12, 15, 36], and illustrates the change from traditional demarcations of professional jurisdiction in favour of interprofessional collaboration between doctors and nurses [37–39]. While we found many examples of this shared decision-making to support timely symptom control, it was not a universal experience, and nurses were mindful not to appear as though they were usurping GPs’ authority or challenging their considerable expertise. This was particularly evident among less experienced nurses or in contexts where GPs were perceived as keen to maintain traditional role boundaries. Cross-disciplinary training for all primary care clinicians, focused on negotiated boundaries and respective expertise [37], along with access to experiential learning with specialists and more experienced peers, could foster more effective collaboration in end-of-life care.

While some of our participants were happy to accommodate the increasing complexity of their roles, particularly where it afforded independence and autonomy in decision-making, this was not the case for all. Participants felt their community end-of-life care work engendered greater professional risk, and the fear of losing their Professional Identification Number (PIN) was ever present in our data. Nurses protected themselves by, for example, refusing to renew syringe drivers alone or strictly adhering to policy and rule-based practices. Perceived professional risk may also have influenced nurse prescribers’ preference that GPs write prescriptions and contributed to non-prescribing nurses’ reluctance to take on this additional responsibility. Bowers and Redsell (2017) also found that nurse prescribers would prefer to ‘share’ the responsibility of prescribing with GPs [13]. Recent UK research suggests that increasing the number of nurse prescribers would free up GP time and reduce healthcare costs [3]. This approach may also support the growing global demand for end-of-life symptom relief [40, 41]. However, for nurses to take on additional responsibilities in end-of-life care such as prescribing, practical changes including access to electronic prescribing systems is needed [21], along with greater understanding of cultures of practice and nurses’ perceptions of regulatory support. Clearer guidance and consistent support from professional nursing organisations, managers and regulatory bodies may help to address nurses’ legitimate concerns and facilitate role expansion.

### Strengths and limitations

Our study included nurses from a range of specialisms and pay bands, with varying levels of clinical experience. This diversity provided a rich account of the varied roles community nurses play in end-of-life care and systems for using injectable medication. However, we were unable to recruit other professionals referenced in nurses’ narratives such as GPs and pharmacists. Similarly, we do not present the perspectives of patients and families in this study regarding nurses’ roles in their end-of-life care, although this is the focus of a forthcoming paper. The study was conducted in two geographical areas in England, encompassing varied communities. Whilst meeting increasing end-of-life care needs by stretched community-based nursing workforces is a global phenomenon [42], our findings are not transferable to all international settings due to differences in healthcare systems, access to controlled drugs, professional roles and regulatory frameworks. While the analysis was largely conducted by a single researcher (RF), key decision points were discussed and refined in collaboration with a second researcher (BB). Further refinement of the findings and interpretations was supported though discussions with our Public and Clinician Advisory Group.

## Conclusion

Our study highlights the growing challenges faced by community nurses in England in providing holistic end-of-life care for patients and their families. Nurses undertake considerable hidden work as they navigate and adapt injectable medication care to ensure timely symptom management. Recognising and addressing this hidden work could inform improvements in systems for prescribing, dispensing and administering injectable medications. As community nurses increasingly assume responsibilities previously held by specialist nurses and GPs, appropriate training, experiential learning opportunities and professional support are essential to ensure they feel confident and capable in these expanding roles. To enable nurses to negotiate increasingly blurred professional boundaries and mitigate legitimate concerns about professional risk, regulatory bodies and healthcare systems must ensure nurses are adequately empowered and protected.

## Supplementary Information

Supplementary Material 1: Healthcare professional interview topic guide (pdf). This document contains a list of instructions, questions and prompts used by BB and RF to guide interviews with healthcare professionals in the study.

## Data Availability

The datasets generated and analysed during the current study are not publicly available due to their sensitive subject nature. However, they can be requested by contacting the corresponding author and may be available to researchers with evidence of a study protocol and Research Ethics Committee approval, and on completion of a data use and sharing agreement.
