# Supplementary material for: Hidden work and blurred boundaries: a qualitative study of how community nurses navigate and adapt injectable medication processes to provide timely end-of-life symptom control
Source: BMC Palliat Care. 2026 May 28;25:222. doi: 10.1186/s12904-026-02159-0 (PMC13425938; doi:10.1186/s12904-026-02159-0)
Supplement: Supplementary file 1 — Supplementary Material 1: Healthcare professional interview topic guide (pdf). This document contains a list of instructions, questions and prompts used by BB and RF to guide interviews with healthcare professionals in the study. [file 12904_2026_2159_MOESM1_ESM.docx]

**Hidden work and blurred boundaries: A qualitative study of how community nurses navigate and adapt injectable medication processes to provide timely end-of-life symptom control**

**Supplementary material file 1 - Healthcare professional interview topic guide**

| **INJECTABLE MEDICATION STUDY**  **INTERVIEW SCHEDULE: HEALTHCARE PROFESSIONAL (DOCTOR, NURSE OR PRAMEDIC)** |
| --- |

Note: 1. Questions will be continually reshaped in response to data from previous interviews and

facets of interest in each patient-centred case.

**2. The questions and prompts selected will depend on the context and experiences shared.**

**Introduction:**

- Introduce yourself.
- Discuss the purpose of the study find out people’s opinions and experiences concerning the use of injectable symptom control medications. These are sometimes called ‘Just in Case drugs’ and are kept in the home in case they are needed. We are interested in what works well and where and how processes / systems for using injectable medications can be improved.
- The focus of the first part of the research conversation will be on a specific patient case, as they are also taking part in the study, and it may help to have their medical records to hand to jog your memory.
- Make the participant feel comfortable!
- Clarify that there are no right or wrong answers. This research is to understand patients, family caregivers, and healthcare professionals’ perspectives and if these are similar or differ.
- We can pause or stop the interviews at any time, please just say if you would like to.
- Please ask for explanations of any questions whenever you want.
- Interviews will be recorded with your permission.
- All participant information collected during the course of the research will be kept strictly confidential. The only situation in which we foresee that confidentiality might be broken

would be if information is identified during the research that causes concern for the welfare of patients or others.

**Explain that there are three broad themes the research conversation is going to cover:**

- How you see your role in looking after terminally ill people
- Your experiences of the prescribing and use of injectable medications and associated conversations in (patient’s name) case
- Your wider experiences of processes / systems for using injectable medications

**Semi-structured interview questions**

*Prompt: turn recorders on*

1. **Can you start by telling me a little about your role in looking after terminally ill people?**
2. **What matters most to you in prescribing / using injectable medications as part of last days of life care at home?**

**Thank you, that is really useful to understand. I would now like to ask about one of the patient cases I am exploring as part of my research. [give a brief overview of the person’s case]**

**NB. DATA FROM QUESTIONS 3-11 NOT USED FOR THE CURRENT ANALYSIS**

1. **Could you give me a resume of your involvement in their case and the situation, including injectable medication prescribing / the use of the injectable medication?**

*Prompts:*

- *Were there any events or changes that prompted the decision to prescribe / use drugs?*
- *Were they prescribed ahead of need or in response to existing symptoms?*
- *In your experience, does anything make decisions to prescribe easier or more challenging?*

1. **Could you tell me about your experiences of any conversations with (patient name) and their family caregiver (family / friend) around prescribing the injectable medications?**

*Prompts:*

- ***(If they were involved) What happened next?***
- *Can you recall the discussion with the patient and / or their family caregiver about prescribing the drugs?*
- ***(If yes)*** *How did it go? – were there any barriers or facilitators to conversations?*
- ***Did anyone have discussions on when and how to access help with symptoms and medications?***
- *Did (patient name) and their family talk to other professionals about the medications or ask further questions about them once they were in the home?*

1. **Do you think (patient name) and their family clearly understood the purpose and significance of the injectable medications (’Just in Case drugs’)?**

*Prompts:*

- *How do you think the patient / family caregiver felt about having these in the home?*

1. **How easy was it to get the prescribed medication dispensed and into the home?**

*Prompts:*

- *Can you recall who did what once the medications were prescribed?*
- *Were any delays or barriers experienced?*
- ***(If unsure)*** *Could you talk me through who does what next with the prescription and permission to administer forms once they have been completed by the prescriber?*

**Go to question 9. if drugs have not been used.**

1. **(If drugs administered) Can you remember why drugs were administered?**

*Prompts:*

- ***Did (patients name) and their family express how they found accessing help?***
- *Who administered the injectable medications?*
- *Which ones and for how long were they given?*
- *Did any events or changes prompt the decision to give the injectable medications?*

1. ***(If drugs administered)* Can you recall if you or other professionals had any conversations about administering the injectable medications with the patient or their family caregiver?**

*Prompts:*

- *What was discussed?*
- *How did it go? – were there any barriers or facilitators to conversations?*
- *If no discussions, were there reasons why they did not happen?*

1. ***(If drugs administered)* Were the patient and their family caregiver involved in the decision to administer the injectable medications?**

*Prompts:*

- ***(If no)*** *Were there reasons why they were not involved in the decision to use the drugs?*
- ***Were there any barriers or facilitators to involving (patients name) and / or their family caregiver in these decisions?***

1. **How do you think the patient / family caregiver felt about having the drugs administered?**

*Prompts:*

- *Do you think the patient and their family caregiver clearly understood the purpose of the injectable medications being administered?*

1. ***(If drugs not administered)* Could you explain the reasons why drugs were not used?** *Prompts:*

- *How long have they been in the home for / how long were they in the home for?*

**Thank you, that really helpful to know.** I’d like to explore your experiences of processes / systems to use injectable medications beyond (patient’s name) case.

1. **How easy do you find the process of prescribing and administering medication?**

*prompts*

- **Are there any barriers to timely prescribing of drugs? – if so, do you do anything to expedite this?**
- **How do patients get the drugs to their homes?**
- **What happens if they live alone? - Do you get involved in obtaining medications for patients?**

1. **What criteria do you use in judging whether to prescribe injectable medications / give an injection?**

*Prompts:*

- Is any aspect physically or cognitively challenging?
- How easy is it to decide the dose to prescribe / administer?

1. **Overall, how well do processes for prescribing, dispensing and using injectable medications perform in your experience?**

***Address Prescribing, dispensing and use separately***

- Are there elements of this system that can be improved in your local area?
- Can you describe the differences in this process during normal hours and out of hours?
- Are there particular challenges out of hours?
- Is anything frustrating with existing processes / systems? Could you give me an example form a case you were involved in
- What would you do improve the current system?
- **What does excellent look like? Could you give me an example from a case you were involved in**
- **Can you think of factors that contribute to things going wrong with the processes / system?**
- **Are there factors that prevent things from going wrong?**

1. **What happens to the injectable medications after a patient’s death?**

*Prompts:*

- **How are they disposed of? - *Do you have any concerns about this process?***
- How do you approach a conversation with the family about drug disposal?
- Do families raise any concerns about doing this?
- **Do you have any way of knowing if medications have been safely disposed of?**
- Who does this?
- What does the team do regarding disposal of medication if the patient lived alone?
- Do you have any concerns about this process?
- **Would you be willing to take drugs back to a pharmacy on behalf of patients/carers?**

1. **Is there anything else you would like to say or pressing thoughts that I have not asked you about?**

***Closing the interview***

Thank them for their participation. **Turn recorder off.**

Reiterate that end of life care can be challenging to talk about. Remind participant that the Participant Information Sheet has the contact details of XXXXXXXXXXX (redacted for anonymity), a nurse with experience of research in studies of ageing and end-of-life care, who is available to discuss any issues that may arise from the research discussion. Participants will be encouraged to speak to colleagues they work with or consult their own GP should they need further support.

**Ask if they would like a copy of the results emailed when they are published – confirm email address.**

***After the interview***

Record anonymised field-notes: observations, feelings, thoughts, reactions about the research conversation and narratives shared
